# Autophagy and Alzheimer’s Disease: From Molecular Mechanisms to Therapeutic Implications

**DOI:** 10.3389/fnagi.2018.00004

**Published:** 2018-01-30

**Authors:** Md. Sahab Uddin, Anna Stachowiak, Abdullah Al Mamun, Nikolay T. Tzvetkov, Shinya Takeda, Atanas G. Atanasov, Leandro B. Bergantin, Mohamed M. Abdel-Daim, Adrian M. Stankiewicz

**Affiliations:** ^1^Department of Pharmacy, Southeast University, Dhaka, Bangladesh; ^2^Department of Experimental Embryology, Institute of Genetics and Animal Breeding, Polish Academy of Sciences, Magdalenka, Poland; ^3^Department of Molecular Biology and Biochemical Pharmacology, Institute of Molecular Biology “Roumen Tsanev”, Bulgarian Academy of Sciences, Sofia, Bulgaria; ^4^Department of Clinical Psychology, Tottori University Graduate School of Medical Sciences, Tottori, Japan; ^5^Department of Molecular Biology, Institute of Genetics and Animal Breeding, Polish Academy of Sciences, Magdalenka, Poland; ^6^Department of Pharmacognosy, University of Vienna, Vienna, Austria; ^7^Department of Pharmacology, Federal University of São Paulo, São Paulo, Brazil; ^8^Department of Pharmacology, Suez Canal University, Ismailia, Egypt; ^9^Department of Ophthalmology and Micro-technology, Yokohama City University, Yokohama, Japan

**Keywords:** autophagy, Alzheimer’s disease, amyloid beta, tau

## Abstract

Alzheimer’s disease (AD) is the most common cause of progressive dementia in the elderly. It is characterized by a progressive and irreversible loss of cognitive abilities and formation of senile plaques, composed mainly of amyloid β (Aβ), and neurofibrillary tangles (NFTs), composed of tau protein, in the hippocampus and cortex of afflicted humans. In brains of AD patients the metabolism of Aβ is dysregulated, which leads to the accumulation and aggregation of Aβ. Metabolism of Aβ and tau proteins is crucially influenced by autophagy. Autophagy is a lysosome-dependent, homeostatic process, in which organelles and proteins are degraded and recycled into energy. Thus, dysfunction of autophagy is suggested to lead to the accretion of noxious proteins in the AD brain. In the present review, we describe the process of autophagy and its importance in AD. Additionally, we discuss mechanisms and genes linking autophagy and AD, i.e., the mTOR pathway, neuroinflammation, endocannabinoid system, *ATG7, BCL2, BECN1, CDK5, CLU, CTSD, FOXO1, GFAP, ITPR1, MAPT, PSEN1, SNCA, UBQLN1*, and *UCHL1*. We also present pharmacological agents acting via modulation of autophagy that may show promise in AD therapy. This review updates our knowledge on autophagy mechanisms proposing novel therapeutic targets for the treatment of AD.

## Introduction

Introduced to biology in 1963 by Belgian biochemist Christian de Duve (De Duve and Wattiaux, 1966) autophagy (from Greek “self-eating”) is an intracellular self-degradative process that is responsible for the systematic degradation and recycling of cellular components such as misfolded or accumulated proteins and damaged organelles (Glick et al., 2010). In 2016, the Japanese cell biologist Yoshinori Ohsumi was awarded Nobel Prize in Physiology or Medicine for identification of autophagy-related genes and the discovery of the mechanisms of autophagy (Nobelprize.org, 2017).

Autophagy has been classified into three categories based on the mechanism by which intracellular constituents are supplied into lysosome for degradation: microautophagy, chaperone-mediated autophagy, and macroautophagy. In microautophagy, the cytoplasmic material is absorbed into lysosome by direct invagination of the lysosomal membrane (Marzella et al., 1981). The chaperone-mediated autophagy facilitates the degradation of cytosolic proteins by directly targeting them to lysosomes and into the lysosomal lumen (Kaushik and Cuervo, 2012). In macroautophagy, degradable contents of cytoplasm are encapsulated in subcellular double-membrane structures named “autophagosomes”. Autophagosomes transport the cell “waste” to the lysosomes for degradation (Settembre et al., 2013). Macroautophagy is the most predominant form of autophagy and will be denoted as such in this review.

Healthy mammalian cells show a low basal level of autophagy (Funderburk et al., 2010). This basal autophagic activity plays a dominant role in the intracellular homeostatic turnover of proteins and organelles (Funderburk et al., 2010). Basal activity of autophagy is essential in post-mitotic neuronal cells, possibly due to their inability to dilute noxious components through cell division (Funderburk et al., 2010). Autophagic activity is enhanced by diverse stresses such as nutrient starvation, hypoxia or inflammation (Melendez and Neufeld, 2008; Francois et al., 2013). Enhanced autophagy participates in various physiological processes and pathological conditions, including cell death, removal of microorganisms invading the cell, and tumor suppression (Glick et al., 2010). On the other hand, reduced autophagic potential is associated with aging (Rubinsztein et al., 2011). During autophagy, proteins are degraded into amino acids, which provide an energy source and are likely used as building blocks for protein synthesis (Onodera and Ohsumi, 2005; Meijer et al., 2015). Thus, dysregulated autophagy may result in accumulation of proteins inside the cell. Various autophagy dysfunctions may contribute to neurodegeneration or neurodegeneration-like symptoms, for example inhibition of the fusion of an autophagosome with a lysosome (Boland et al., 2008), reduction of lysosomal acidification (Shen and Mizushima, 2014) or accumulation of proteins in cells (Garcia-Arencibia et al., 2010).

Alzheimer’s disease is the most predominant type of dementia diagnosed in the aged people (Uddin et al., 2016). It is characterized by a chronic, irreversible, and progressive neuronal degradation in the human brain caused by complex pathophysiological processes, including oxidative stress, neuroinflammation, excitotoxicity, mitochondrial dysfunction, proteolytic stress, and more (Jellinger, 2010). Formation of intracellular NFTs and extracellular senile plaques in the brain are two common hallmarks of AD (Armstrong, 2009). NFTs consist of aggregated, abnormally hyperphosphorylated MAPT (Iqbal et al., 2010). Senile plaques are primarily composed of insoluble and toxic amyloid-β (Aβ) peptides and of dysfunctional dystrophic neurites, which include abnormally large amounts of neurofilament, tau, or chromogranin A proteins (Dickson et al., 1999; Armstrong, 2009).

Despite the accumulated wealth of knowledge, AD remains incurable. The significance of autophagy in pathophysiology of AD is now appreciated due to the discoveries of molecular mechanisms for autophagy. The objective of this review is to introduce an outline of the discovery of autophagy and describe the relationship between autophagy and AD.

Please consider, that in the present review the names of genes are written in italic, while names of proteins are written in standard font. Names of human or *Saccharomyces* sp. genes/proteins are written in all capital letters. Names of rodent genes/proteins are written in capital letter followed by small letters.

## History of Autophagy Research

### Lysosome

In the mid 1950’s researchers explored a novel specialized cellular substructure (organelle), encapsulating enzymes that digest macromolecules such as proteins and lipids (Xu and Ren, 2015). This compartment was named “lysosome” (de Duve, 2005). The lysosome was discovered by the Belgian cytologist and biochemist Christian de Duve. For this achievement de Duve was awarded the 1974 Nobel Prize in Physiology or Medicine (Blobel, 2013).

The lysosome is generally 100–1500 nanometers in diameter and enclosed by a typical lipid bilayer membrane (Xu and Ren, 2015). Lysosomes contain more than 60 different hydrolase enzymes such as proteases and lipases (Xu and Ren, 2015). The lysosomal enzymes are the most active in acidic environment, such as this in the lumen of a lysosome (pH of approximately 4.6) (Xu and Ren, 2015). This characteristic of lysosomal enzymes provides protection against unrestrained, pathological digestion of the constituents of the cell, as cytosol pH is almost neutral (pH 7.2) (Alberts et al., 2002). Hence, even if lysosomal membrane would become damaged and the enzymes were to leak into the cytosol, harm to the cell itself would be minimal (Alberts et al., 2002).

Lysosomes serve as an intracellular digestive system protecting the cell from its unused and/or noxious constituents (Huber and Teis, 2016). Furthermore, lysosomes are involved in various cell processes, including secretion, cell membrane repair, cell signaling and energy metabolism (Settembre et al., 2013). Mutations in the genes involved in the synthesis of lysosomal proteins have been linked to over 40 human genetic diseases (lysosomal storage diseases) (Parenti et al., 2013).

### Proteasome

Like autophagy, the ubiquitin-proteasome system is another degradation pathway for cellular proteins. During the 1970’s and 1980’s, researchers began to study second system of cell protein degradation, namely the “proteasome”. The significance of intracellular proteolytic degradation and the contribution of ubiquitin-proteasome system to the proteolytic pathways (i.e., discovery of ubiquitin-mediated proteolysis) was acknowledged with the award of the Nobel Prize in Chemistry in 2004 to the Israeli biologist Aaron Ciechanover; the Hungarian-born Israeli biochemist Avram Hershko and the American biologist Irwin Rose (Karigar and Murthy, 2005).

Proteasomes are large, multisubunit protease complexes that are responsible for the degradation of unnecessary or damaged proteins by proteolysis (Tanaka et al., 2004). Proteasomal degradation produces amino acids, which may be subsequently used in generation of new proteins (Rogel et al., 2010). Proteins are labeled for degradation with a 76-amino acid protein called “ubiquitin” (Weissman, 2001). Single labeling event leads to a cascade, resulting in the formation of polyubiquitin chain, which binds to the proteasome for proteolysis (Ciechanover and Schwartz, 1998; Li and Ye, 2008).

The proteasomal degradation pathway plays an important role in numerous cellular processes, for example cell cycle and immune response (Ciechanover and Schwartz, 1998). Improper ubiquitin-mediated protein degradation has been linked to several neurodegenerative disorders including AD, Parkinson’s disease, Huntington’s disease and amyotrophic lateral sclerosis (Atkin and Paulson, 2014).

Recent studies showed the existence of cross-talk between proteasomal and autophagy pathways (Lilienbaum, 2013). Both processes share protein degradation signaling network molecules, may be recruited by ubiquitinated substrates, and under specific conditions display compensatory functions to maintain cellular homeostasis (Lilienbaum, 2013).

### Autophagosome

Additional biochemical and microscopic investigations identified a new type of vesicles carrying cellular cargo to the lysosome for degradation. Christian de Duve, the discoverer of the lysosome, introduced the term “autophagy” to define this process (Klionsky, 2008). The new vesicles were named autophagosomes (Klionsky, 2008). Autophagy research was kick-started in 1990s with studies performed by Yoshinori Ohsumi, for which he was awarded the 2016 Nobel Prize in Physiology or Medicine (Nobelprize.org, 2017).

He studied autophagy using as a model organism the budding yeast (Takeshige et al., 1992), whose vacuole is functionally similar to the mammalian lysosome (Li and Kane, 2009). His group has shown that starved yeast devoid of some of the functional vacuolar proteases developed spherical bodies inside the vacuoles (Takeshige et al., 1992). These bodies were encompassed by a membrane and contained constituents of cytosol such as cytoplasmic ribosomes, mitochondria, rough endoplasmic reticulum fragments, glycogen, etc. The constituents would be normally degraded in yeast cultured on the nutrient-poor medium to facilitate adaptation to adverse environment. Without functional proteases the degradation could not commence, and so the spherical bodies remained easily perceivable. These spherical structures were named “autophagic bodies”.

**FIGURE 1:**
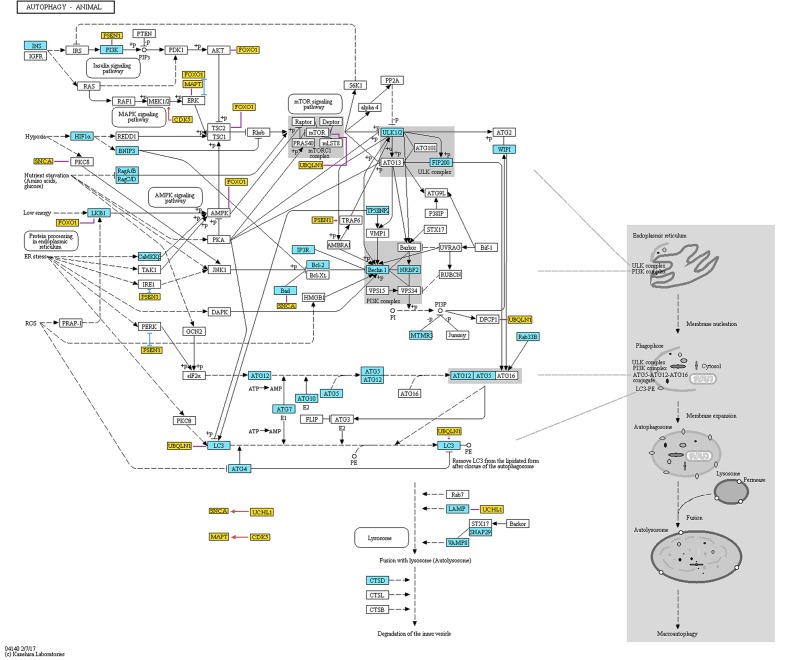
Representation of proteins and protein complexes involved in the “Autophagy – animal” KEGG pathway. This figure was taken from the KEGG database (http://www.genome.jp/kegg-bin/show_pathway?ko04140) and modified. Blue boxes mark the proteins that are associated with AD. Orange boxes mark additional proteins that are not originally included in the pathway. These genes are associated with both AD and autophagy, and are discussed in the present review. Red, blue, and violet lines mark partners with which the additional proteins interact (red color means activation, blue color means inhibition, and violet color means unspecified or complex (e.g., both inhibitory and stimulatory effect) according to STRING database). The interactions data were extracted from the STRING database (http://string-db.org). To assure that the presented data is reliable, we have included only interactions that showed at least medium STRING confidence score and were either identified in an experiment or are annotated in manually curated databases. Additionally, we have added interaction between GFAP and LAMP, which was not included in STRING database but was found by manual literature search. Permission to use KEGG figure was granted.

In 1993, Ohsumi’s group published research, in which they identified 15 genes (*APG1-15)* that are essential for the activation of autophagy in yeast cells (Tsukada and Ohsumi, 1993). Later, as a result of efforts of the scientific community to standardize the gene names, the *APG* genes were renamed to *ATG* (Klionsky et al., 2003). Afterward, Ohsumi’s group cloned numerous *ATG* genes and identified the function of their protein products (e.g., Funakoshi et al., 1997; Matsuura et al., 1997). Further studies established the interactions between these products providing the basis for autophagy mechanisms (see **Figure 1**). They found that the ATG1 protein (now: ULK1) combines with the product of the *ATG13* gene to form autophagic complex (Kamada et al., 2000). This process is controlled by target of rapamycin (TOR) kinase (Kamada et al., 2000). Further, Ohsumi’s group established that for proper activation the ATG1 protein needs to form complex not only with ATG13, but also with ATG17 (RB1CC1/FIP200) (**Figure 1**) (Ohsumi, 2014). As shown in **Figure 1**, the formation of this complex is the first stage in autophagosome genesis (The Nobel Assembly at Karolinska Institutet, 2016). The phosphatidylinositol-3 kinase (PI3K) complex that is composed of PIK3C3 (VPS34), PIK3R4 (VPS15), BECN1, and ATG14 (Barkor) proteins (Ohsumi, 2014), produces phosphatidylinositol-3 phosphate (PtdIns3P or PI3P), which facilitates binding of further effector proteins to the membrane of the autophagosome (Ohsumi, 2014).

In the late 1990’, Ohsumi’s group discovered two ubiquitin-like conjugation systems involved in the autophagosome formation (**Figure 1**) (Ohsumi, 2014). First conjugation system results in a formation of an ATG12-ATG5 complex, while the second one results in the formation of a conjugate of ATG8 (MAP1LC3A/GABARAPL2/LC3) with a membrane phospholipid, phosphatidylethanolamine (Ohsumi, 2014). The formation of both conjugates is mediated by the ATG7 protein (Ohsumi, 2014). ATG12-related system regulates ATG8 lipidation and lipidated ATG8 is a crucial participant in the processes of autophagosome elongation (Nakatogawa et al., 2007; Nakatogawa, 2013). These two conjugation systems are evolutionary conserved among yeast and mammals (Ohsumi, 2014). Actually, fluorescently labeled product of the mammalian homologue of yeast gene *ATG8* is used as an indicator of the formation of autophagosome in mammalian systems (Kabeya et al., 2000; Mizushima et al., 2004).

The *ATG* genes proved to play crucial roles in mammalian organisms. For example, mice with knock-out of *ATG5* gene die in the first days of life due to their inability to cope with the post-labor starvation period (Kuma et al., 2004). In this life period, functional autophagy allows the neonate to keep the steady energy supply before milk feeding starts (Kuma et al., 2004). Further studies on knockout mouse models lacking functional versions of autophagy-related genes have established the functions of the autophagy in different mammalian tissues (Mizushima and Komatsu, 2011).

## Biological Mechanisms Linking Autophagy and AD

### Aβ Metabolism and the Autophagy

Alzheimer’s disease is a progressive neurodegenerative disorder, which pathophysiology includes formation of Aβ aggregates (Oddo et al., 2006). In a healthy human central nervous system the production rate of Aβ peptides is generally lower than their rate of clearance, at 7.6 and 8.3% per hour, respectively (Bateman et al., 2006).

Autophagy is a key regulator of Aβ generation and clearance (Nilsson and Saido, 2014). Aβ peptides are produced through cleavage of amyloid precursor protein (APP) in the autophagosomes during autophagic turnover of APP-rich organelles (Nixon, 2007; Steele et al., 2013). In AD the maturation of autophagolysosomes (i.e., autophagosomes that have undergone fusion with lysosomes) and their retrograde passage toward the neuronal body are hindered (Nixon, 2007). This contributes to an immense accretion of autophagic vacuoles in neurons. Such accretion may be related to dysfunction of the ESCRT-III complex. This dysfunction is associated with neurodegeneration (Lee et al., 2007; Yamazaki et al., 2010) and may affect autophagosome maturation by disrupting fusion of autophagosomes with the endolysosomal system (Rusten and Stenmark, 2009).

There are two pathways for disposing Aβ peptides. Firstly, they can be simply degraded by various Aβ-degrading proteases, including BACE1 and CTSD (Saido and Leissring, 2012). Secondly, Aβ peptides can accumulate in autophagosomes of dystrophic neurites (i.e., main constituents of neuritic senile plaques in AD), thus being incorporated into primary intracellular reservoir of toxic peptides (Nixon et al., 2005; Yu et al., 2005). The second recycling path of Aβ peptides is especially prevalent in the brains of people suffering from AD (Nilsson et al., 2013; Nilsson and Saido, 2014).

A paper published by Nilsson et al. (2013) shows that Aβ peptides are released from neurons in an autophagy-dependent manner and suggests that the accumulation of intracellular Aβ plaques is toxic to brain cells leading to AD pathology. To explore the role of autophagy in Aβ pathology *in vivo*, Nilsson et al. (2013) crossed *App* transgenic mice, carrying Swedish mutation, with mice lacking functional autophagy mechanisms in the forebrain neurons due to conditional knockout of *Atg7*. They observed that the offspring had far fewer extracellular Aβ plaques than the mice with functional autophagy. The decrease of extracellular Aβ plaque content reported by Nilsson et al. (2013) was caused by inability of cells with disrupted autophagy to secrete Aβ peptides. Indeed, they report that in the autophagy deficient mice, reduction in Aβ peptides secretion co-occur with accumulation of Aβ inside the brain cells (Nilsson et al., 2013). Moreover, in the autophagy deficient mice, intracellular aggregation of Aβ likely caused neurodegeneration and, together with amyloidosis, memory impairment (Nilsson et al., 2013). These findings are in agreement with previous reports that intracellular Aβ is neurotoxic (Zhang et al., 2002).

Summing up, impaired autophagy is a well-established participating mechanism in the pathology of Aβ metabolism of AD.

### Neuroinflammation

Present knowledge suggests that inflammation, autophagy and AD are connected processes. A study by Francois et al. (2013) provided an example of cross-talk between them. They showed that Aβ42 influences the expression and activation of some proteins involved in autophagy (p62, p70S6K) *in vitro* (Francois et al., 2013). They also showed that the processes of inflammation and autophagy interact within brain cells, as severe inflammation induced by IL-1β activated autophagy in microglia grown in tri- or mono-cultures (Francois et al., 2013). Although the role of IL-1β itself in AD is unclear, we do know how the neuroinflammation contributes to AD pathogenesis (Zhang and Jiang, 2015), and why IL-1β is a key mediator of neuroinflammation (Basu et al., 2004). Hence, one could speculate that IL-1β may play role in pathogenesis of AD by eliciting both neuroinflammation and autophagy. It seems viable that during the course of AD, immune signals induce autophagy. Indeed, it was shown that neuroinflammation might influence autophagy following stress-induced hypertension (Du et al., 2017). Correspondingly, another study reported that adult mice bearing mutations of *App* and *Psen1* genes showed higher brain levels of inflammatory mediators (including Il-1β) along with accumulation of autophagic vesicles within dystrophic neurons in the cortex and hippocampus (Francois et al., 2014). Moreover, the levels of inflammatory mediators correlated with expression of key autophagy regulators such as mTOR and Becn1 (Francois et al., 2014). On the other hand, Ye et al. (2017) suggest, that inhibition of autophagy may enhance microglia activity, including secretion of cytokines such as Il-1β and generation of toxic reactive oxygen species (ROS) *in vitro*.

Taken together, these studies suggest that AD and neuroinflammation feed autophagy (and each other), while autophagy decreases inflammation in the brain. Thus, the increase in autophagy may play some protective role during the course of AD via interaction with the immune system.

### Mechanistic Target of Rapamycin (mTOR) Pathway

Mechanistic target of rapamycin signaling pathway is initiated by nutrients and growth factors and regulates autophagy (Jung et al., 2010). Human studies suggest participation of mTOR signaling in AD (Sun et al., 2014). It has been shown that mTOR signaling is inhibited in cortex and hippocampus of adult AD model mice (Francois et al., 2014). Decreased mTOR signaling leads to reduction in levels of Aβ (Spilman et al., 2010; Caccamo et al., 2014) and protects memory of AD model mice from deterioration (Caccamo et al., 2014). A study performed by Spilman et al. (2010) on mouse model of AD reported that blocking the mTOR signaling with rapamycin relieves cognitive deficits and reduces amyloid pathology, likely by activating autophagy in brain cells. Correspondingly, studies show that diet enriched with rapamycin prolongs lifespan of animals (Harrison et al., 2009). This may be relevant to AD research, because age is a major factor in the pathogenesis of AD (Guerreiro and Bras, 2015). Moreover, studies on human cells have shown that mTOR mediates intra- and extra-cellular distribution of tau (Tang et al., 2015), its phosphorylation and accumulation as well as resulting behavioral effects of tau pathology (Caccamo et al., 2013). Finally, multiple compounds tested for their efficacy as AD medication impose their beneficial effect by inducing mTOR-depending autophagy (see below).

Summarizing, mTOR pathway is currently one of the most promising targets for autophagy-related AD therapy.

### Endocannabinoids

Recently published reports highlight the role of the endocannabinoid system in neurodegenerative diseases and autophagy (Maroof et al., 2013; Shao et al., 2014; Bedse et al., 2015). Endocannabinoids are lipophilic molecules that, when released, activate the cannabinoid receptors CNR1 and CNR2 (cannabinoid receptor 1 and 2) (Katona and Freund, 2012).

Mice with a *Cnr1* deletion have shown a pathological accumulation of some proteins, which are not degradable by lysosomal enzymes through autophagy (Piyanova et al., 2013). Knockdown of CNR1 expression by siRNA results in both mTOR- and BECN1-independent increase of autophagic vesicle formation (Hiebel et al., 2014).

In a human AD frontal cortex, expression of the CNR1 receptor was significantly reduced (Ramirez et al., 2005; Solas et al., 2013). In an AD mouse model Cnr1 was decreased in dorsal hippocampus and basolateral amygdala complex (Bedse et al., 2014). It seems that in frontal cortex and hippocampus the activity of the CNR1 receptor depends on the progression of AD. While in early AD the activity is increased, it shifts to attenuation in later AD stages (Manuel et al., 2014). Additionally, the expression levels of the CNR2 receptor were increased in microglia cells of an AD patient’s in the hippocampus, entorhinal cortex and frontal cortex (Benito et al., 2003; Solas et al., 2013). The high expression of CNR2 receptor was correlated with the Aβ42 levels and senile plaque burden (Solas et al., 2013).

All these findings suggest that there is a non-trivial connection between endocannabinoids, autophagy, and AD. A further investigation is required to fully understand the mechanisms involved.

### Genes Common to Autophagy and AD

**FIGURE 2:**
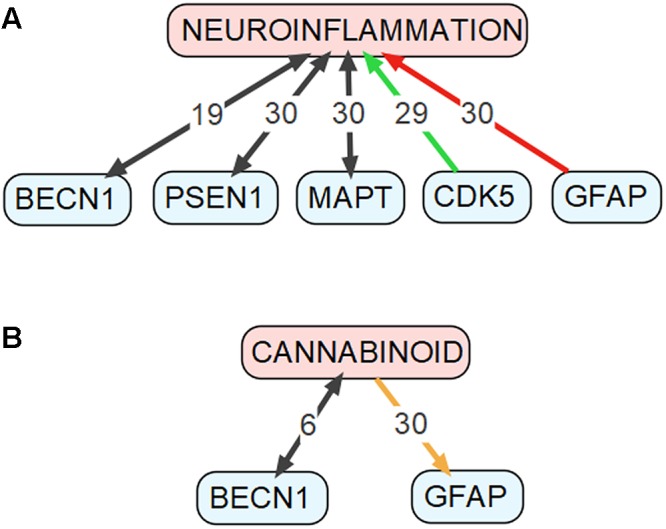
Connections between genes discussed in the “Genes Common to Autophagy and AD” section and **(A)** neuroinflammation as well as **(B)** cannabinoids. This figure was drawn based on data obtained using the Chilibot tool. Black arrows mark relationships that are neither obviously stimulatory nor inhibitory. Orange arrow marks both stimulatory and inhibitory relationship. Red arrow marks inhibitory relationship. Green arrow mark stimulatory relationship. The respective numbers mark the weight of the relationship according to the Chilibot tool.

To identify the genes that may mediate cross-talk between molecular mechanisms of autophagy and AD, we have compared two groups of genes: (1) genes involved in autophagy, defined as being included either in Gene Ontology term “autophagy” (GO:0006914, *Homo sapiens*) or in KEGG Pathway (Kanehisa et al., 2017) “autophagy-animal” (ko04140), and (2) genes involved in AD, defined as being included either in databases AlzBase (Bai et al., 2016) or AlzGene (Bertram et al., 2007), or related to AD as shown by the text-mining tool GLAD4U (Jourquin et al., 2012). AlzBase provides data on “gene dysregulation in AD and closely related processes/diseases such as aging and neurological disorders” (Bai et al., 2016), while AlzGene provides data on “genetic association studies in the field of AD” (Bertram et al., 2007). AlzGene can be treated as a comprehensive database of genes that were associated with AD before year 2011, when it was last updated. Unfortunately, currently there is no other database that collects such information. Finally, GLAD4U is a prioritization tool querying PubMed for given phrase and returning associated genes (Jourquin et al., 2012). The genes that are common to both groups’ are summarized in Supplementary Table S1. For detailed discussion we selected genes, which met following requirements: (1) reported to be involved in both autophagy and AD according to the PubMed database, AND (2) constituted top five results from either AlzBase, AlzGene or GLAD4U. Additionally, we arbitrarily selected five genes involved in KEGG Pathway “autophagy-animal” for further discussion. Gene hierarchy was established for AlzBase and AlzGene based on the total number of entries into database and for GLAD4U as a confidence score provided by the tool. Generally, selected genes showed strong (weight > 5) relationship with neuroinflammation, as detected by Chilibot (Chen and Sharp, 2004), especially BECN1, PSEN1, MAPT, GFAP, and CDK5 (see **Figure 2A**). Simultaneously, the genes were not significantly related to the endocannabinoid system (queried in Chilibot via keyword “cannabinoid”), with only BECN1 and GFAP showing strong interaction (see **Figure 2B**). The genes described below were also added to **Figure 1** along with their known interactions with other molecules of the pathway (see also Supplementary Table S2), as extracted from STRING database (organism: *Homo sapiens*) (Szklarczyk et al., 2017).

#### Autophagy-Related 7 (*ATG7*)

As stated previously, *ATG7* is a key gene regulating autophagic conjugation systems (Ohsumi, 2014). *ATG7* is involved in memory functions as evident from a study, in which forebrain-specific *Atg7* knockout mouse have shown memory deficits (Inoue et al., 2012). We have found two studies connecting dysregulated expression of ATG7 protein and AD-like pathology. Decreased levels of the Atg7 protein were found in cerebral cortex and hippocampus of mouse model of AD (Carvalho et al., 2015). On the other hand, no dysregulation of protein expression of ATG7 was found in temporal cortices of AD patients (Crews et al., 2010).

*Atg7* mediates the transport of Aβ peptides to the multivesicular body and their secretion in mouse neurons (Nilsson et al., 2015). Inhibition of *ATG7* expression using siRNA partially protected against increase in production and secretion of Aβ40 *in vitro* (Cho et al., 2015). On the other hand, intra-hippocampal infusion of Aβ is able to increase the expression of the Atg7 protein in hippocampus of rats while reducing their memory performance (Mohammadi et al., 2016).

*ATG7* seems to be involved in degradation of tau. Forebrain-specific *Atg7* knockout in mice resulted in an accumulation of phosphorylated tau protein in hippocampus and cerebral cortex, as well as neurodegeneration evident in loss of hippocampal neurons and memory dysfunction (Inoue et al., 2012).

#### BCL2

BCL2 is an anti-apoptotic factor that interacts with BECN1 to regulate autophagy (Decuypere et al., 2012).

Overexpression of neuronal *Bcl2* improved place recognition memory in mice (Rohn et al., 2008). Contrary, negative correlation between the cortical BLC2 protein expression and memory (immediate recall) was established in AD patients (Perez et al., 2015). Upregulation of the BCL2 protein was found in precuneus (cortex) of AD patients (Perez et al., 2015).

Aβ treatment decreases the BCL2 expression *in vitro* (Clementi et al., 2006), while *APP* mutation (Swedish) mediates similar effect *in vitro* during starvation (Yang et al., 2009). Overexpression of Bcl2 protects against Aβ-related death of neuronal cells *in vitro* (Ferreiro et al., 2007). Rohn et al. (2008) reported that AD model mice engineered to overexpress Bcl2 protein showed decreased processing of App and number of extracellular deposits of Aβ, as compared to base strain (3xTg-AD).

The overexpression of Bcl2 affects also tau processing, reducing the number of NFTs (Rohn et al., 2008).

#### Beclin 1 (*BECN1/ATG6*)

BECN1 protein mediates the initiation of autophagy and genesis of autophagosomes. Becn1 heterozygotic mice (Becn1+/-) show decreased autophagy in neurons (Pickford et al., 2008).

Several reports suggest, that BECN1 is involved in the pathophysiology of AD. Postmortem midfrontal cortex and isolated microglia of AD patients show reduced content of BECN1 protein (Pickford et al., 2008; Lucin et al., 2013). Similarly, reduced Becn1 expression was found in cortex and hippocampus of adult mouse model of AD (Francois et al., 2014). BECN1 may protect against AD-associated cellular death. Xue et al. (2013) report that expression of Becn1 correlates with viability of cells treated with toxic Aβ42. Interestingly, Becn1 activity seems to be regulated by Aβ42 (Nah et al., 2013).

A study performed on the frontoparietal cortex and the hippocampus of mice showed that decreasing of Becn1 expression leads to increased levels of Aβ (Pickford et al., 2008). Becn1-mediated decrease in autophagy leads to accretion of Aβ peptides and, finally, to neurodegeneration (Pickford et al., 2008).

BECN1 is also involved in neuroinflammation and cannabinoid system activity. Inhibition of Becn1 expression increases microglia inflammatory response (Zhou et al., 2011). Chronic LPS-induced inflammation decreases hippocampal Becn1 expression (Jiang et al., 2017). On the other hand, *Cb2r* deletion decreases Becn1 expression in the spinal cord of mice (Shao et al., 2014).

#### Cyclin Dependent Kinase 5 (CDK5)

CDK5 is an autophagy-regulating kinase (Wong et al., 2011), which expression is enriched in central nervous system as shown in Human Protein Atlas (HPA) (Uhlen et al., 2015).

*Cdk5* modulates various cognition-related biological processes such as neurogenesis in adult hippocampus (Crews et al., 2011) and synaptic functions (Sheng et al., 2016). Silencing of hippocampal Cdk5 expression using RNAi resulted in improved memory performance in AD model mice (Posada-Duque et al., 2015). Study connected *CDK5*-associated polymorphisms with increased risk of AD (Rademakers et al., 2005). CDK5 protein expression is enhanced in frontal cortices of AD patients (Sadleir and Vassar, 2012). On the contrary, CDK5 protein expression is decreased in cerebrospinal fluid (CSF) of AD patients (Olah et al., 2015).

CDK5 influences the metabolism and effects of Aβ. CDK5 may regulate BACE1 protein expression (Sadleir and Vassar, 2012) as well as activity (Song W.J. et al., 2015). *BACE1* gene encodes β-secretase, which is a crucial enzyme involved in APP metabolism (Cai et al., 2015). Furthermore, Cdk5 participates in cytotoxic activity of Aβ42 in primary cortical neurons (Chang et al., 2012), mediates Aβ peptide-induced dendritic spine loss (Qu et al., 2011) and APP phosphorylation (Iijima et al., 2000). On the other hand, Aβ increases Cdk5 activity in primary cortical neurons (Seyb et al., 2007).

CDK5 is similarly involved in tau metabolism. Cdk5 binds to tau *in vitro* and is co-localized with it in rat cortex (Li et al., 2006). Cdk5 participates in tau phosphorylation (Noble et al., 2003), although whether this may lead to formation of NFTs is disputed (Bian et al., 2002; Noble et al., 2003). Prevention of Cdk5 hyperactivity in the mouse model of AD protects against tau hyperphosphorylation, Aβ accumulation, memory loss, and enhanced neuroinflammation (Shukla et al., 2013).

#### Clusterin (*CLU/APOJ*)

CLU is a chaperone protein that participates in autophagosome biogenesis via interaction with ATG8E (MAP1LC3A) (Zhang F. et al., 2014).

*CLU* is one of the top AD candidate genes with the third lowest *p*-value of the association (*p* = 3.37E-23) according to the meta-analysis included in AlzGene database (Bertram et al., 2007). Meta-analyses showed the involvement of *CLU*-related mutations in AD pathogenesis (Liu et al., 2014; Shuai et al., 2015). *CLU* mutations that are suggested as causal for AD affect hippocampal connectivity (Zhang et al., 2015), white matter integrity in several brain regions (Braskie et al., 2011), cortical gray matter volume (Stevens et al., 2014), as well as working memory (Stevens et al., 2014) and episodic memory performance (Barral et al., 2012). *CLU* mRNA is upregulated in hippocampi of AD patients (May et al., 1990). According to Miners et al. (2017) CLU protein rises in several brain regions, including frontal cortex, of AD patients in correlation with noxious Aβ40/42 levels. Results of study by Baig et al. (2012) did not confirm these findings. The CLU protein is upregulated in CSF of AD patients (Deming et al., 2016). The content of CLU protein in the blood plasma of AD patients was reported to be dysregulated in some studies (Mullan et al., 2013), while others did not confirm this finding (Deming et al., 2016).

Moreover, CLU protein interacts with Aβ, reduces its aggregation and protects against its toxic effects (Beeg et al., 2016). CLU decreases the Aβ intake by human primary glia cells (Mulder et al., 2014).

The interaction between tau and CLU is less studied (Zhou et al., 2014). However, Zhou et al. (2014) reported that the Clu protein is upregulated in a tau-overexpressing mouse model of AD. Furthermore, the AD-associated *CLU* polymorphism rs11136000 regulates the levels of tau protein in CSF in AD patients (Zhou et al., 2014).

#### Cathepsin D (*CTSD*)

Cathepsin D is a lysosomal protease (Dean, 1975) that is involved in degradation of the APP protein (Letronne et al., 2016).

Two meta-analyses on the influence of *CTSD* mutation rs17571 on AD yielded contrary results (Schuur et al., 2011; Mo et al., 2014). Similar discrepancy is also reported for another *CTSD* mutation (Ala224Val) (Ntais et al., 2004; Paz-Y-Miño et al., 2015). Directionality of the change of *CTSD* gene expression seems to depend on studied tissue. CTSD level was decreased in bone marrow-derived monocytes isolated from AD patients (Tian et al., 2014). *CTSD* mRNA expression was upregulated in whole blood of AD patients (Bai et al., 2014). On the other hand, *CTSD* is downregulated on both mRNA and protein levels in skin fibroblasts from AD patients (Urbanelli et al., 2008).

Cathepsin D participates in processing of Aβ peptides (McDermott and Gibson, 1996) and clearance of amyloid plaques *in vitro* (Tian et al., 2014). Nevertheless, Aβ processing mechanisms are fairly resistant to modest (38%) changes in expression of *Ctsd*, at least in cerebral cortex of mouse model of AD (Cheng et al., 2017).

Cathepsin D also interacts with tau protein. Previously mentioned rs17571 mutation causes changes in processing of tau, but not of APP (Riemenschneider et al., 2006).

#### Forkhead Box O1 (*FOXO1*)

*FOXO1* gene encodes transcription factor that plays a role in autophagy modulation in neurons (Xu et al., 2011). *FOXO1* mutation rs7981045 was associated with response of AD patients to a treatment based on acetylcholinesterase inhibitors (Paroni et al., 2014)

#### Glial Fibrillary Acidic Protein (*GFAP*)

GFAP is a cytoskeletal intermediate filament-III and a marker of astrocytes (Sofroniew and Vinters, 2010; Yang and Wang, 2015). GFAP binds with LAMP2A (**Figure 1**) (Bandyopadhyay et al., 2010). Multiple studies found increased levels of GFAP in tissues of AD patients. GFAP levels are increased in the frontal cortices, hippocampi (Korolainen et al., 2005; Kamphuis et al., 2014), and the CSF of AD patients (Ishiki et al., 2016). Moreover, *Gfap* expression is modulated by cannabinoid receptor 1 (Cnr1) in the hypothalamus of mice (Higuchi et al., 2010) and neuroinflammation regulates astrogliosis (abnormal increase in the number of astrocytes) (Carson et al., 2006).

#### Inositol 1,4,5-Trisphosphate Receptor Type 1 (*ITPR1/IP3R1*)

*ITPR1* gene encodes intracellular receptor mediating calcium release from the endoplasmic reticulum (Santulli and Marks, 2015) and also plays a role in inducing autophagy (Messai et al., 2014). Engineered downregulation of *Itpr1* expression protected AD model mice from Aβ accumulation, tau hyperphosphorylation, as well as from dysfunction of memory and hippocampal LTP (Shilling et al., 2014).

#### Microtubule Associated Protein Tau (*MAPT/TAU*)

*MAPT* gene encodes tau protein, which pathology is one of the most well-recognized markers of AD. Autophagy is a main pathway of degradation of tauDeltaC, which is a form of the protein found in the brains of AD patients (Dolan and Johnson, 2010). Autophagy dysfunction plays important role in tau aggregation (Inoue et al., 2012). Tau may also regulate autophagy (Pacheco et al., 2009), likely via inhibition of HDAC6 activity (Perez et al., 2009). Finally, Mapt deficiency reduces neuroinflammation (Maphis et al., 2015), while neuroinflammation in turn induces Mapt phosphorylation (Bhaskar et al., 2010).

#### Presenilin 1 (*PSEN1*)

PSEN1 protein is a regulator of the APP-cleaving γ-secretase complex (De Strooper et al., 1998), and autophagic proteolysis (Neely and Green, 2011).

*PSEN1* gene mutations contribute to the pathogenesis of early onset AD (Karch and Goate, 2015), and this effect may be mediated by loss of stability and hydrophobicity of the proteins encoded by the mutated variants (Somavarapu and Kepp, 2016). CSF of AD patients with *PSEN1* mutations showed lower levels of Aβ than AD patients without *PSEN1* mutation (Ikeda et al., 2013). This may suggest that the proteins are retained in the brain cells due to dysregulated autophagy. Cataldo et al. (2004) compared brains of AD patients with mutation of presenilin 1 with brains of sporadic AD patients. They concluded that *PSEN1* mutation is associated with higher prevalence of lysosomal pathology in neurons of AD patients (Cataldo et al., 2004). This corresponds to report by Lee et al. (2010), where the authors show that *Psen1* is crucial for modulating lysosome acidification and proteolysis during autophagy. Dysregulated lysosomal proteolysis may lead to accumulation of proteins and cell death (Lee et al., 2010). Additionally, PSEN1 is hypothesized to be involved in brain immune response as *Psen1/2* knock-out changes the expression of neuroinflammation-related genes (Mirnics et al., 2008).

#### Alpha-Synuclein (*SNCA/PARK1/NACP*)

Expression of *SNCA* is enriched in brain according to Human Protein Atlas (Uhlen et al., 2015). SNCA regulates autophagosome formation (Yan et al., 2014), but it is also negatively regulated by autophagy (Colasanti et al., 2014).

*SNCA* mutations are connected to the risk of AD (Matsubara et al., 2001; Wang et al., 2016). Changes in expression of SNCA proteins were also reported in some brain regions of AD patients (Quinn et al., 2012). Dysregulated levels of SNCA in CSF are associated with cognitive performance (Korff et al., 2013). Effect of Snca protein expression on memory was also reported in mice (Larson et al., 2012).

SNCA is an important component of Aβ plaques (Ueda et al., 1993). Snca induces expression of Aβ peptides and vice versa (Majd et al., 2013). SNCA also likely regulates APP processing by modulating the activity of BACE1 (Roberts et al., 2017), binds Aβ peptides and promotes their aggregation (Yoshimoto et al., 1995). There are also reports of Snca inhibiting Aβ plaque formation (Bachhuber et al., 2015). On the other hand, Aβ40 decreases SNCA uptake by neurons (Chan et al., 2016).

Similarly to interaction of SNCA with Aβ peptides, SNCA and tau also induce each other fibrillization (Giasson et al., 2003). SNCA binds, phosphorylates, and inhibits microtubule assembly activity of tau (Oksman et al., 2013; Oikawa et al., 2016).

#### Ubiquilin 1 (*UBQLN1*)

*UBQLN1* gene encodes ubiquitin-like protein involved in autophagosome–lysosome fusion (N’Diaye et al., 2009) likely by interacting with ATG8E (MAP1LC3A) (Rothenberg et al., 2010).

There is a strong evidence for involvement of *UBQLN1* in AD pathology. UBQ-8i polymorphism of *UBQLN1* was associated with increased risk of AD in two separate meta-analyses (Zhang and Jia, 2014; Yue et al., 2015). In hippocampi of AD patients UBQLN1 protein localizes to dystrophic neurites (Satoh et al., 2013). Expression of UBQLN1 protein is reduced in temporal and frontal cortices of AD patients (Stieren et al., 2011; Natunen et al., 2016). This decrease may cause enhanced processing and intracellular trafficking of APP (Hiltunen et al., 2006; Stieren et al., 2011), and secretion of Aβ40/42 (Hiltunen et al., 2006).

Moreover, UBQLN1 interacts with BACE1, which is a key APP processing protein. Ubqln1 overexpression causes an increase of Bace1 in neuron-microglia co-cultures, though this effect did not reach significance in the brains of mice (Natunen et al., 2016).

#### Ubiquitin C-Terminal Hydrolase L1 (*UCHL1*)

UCHL1 is a brain-enriched ubiquitin-specific hydrolase (Uhlen et al., 2015). It influences autophagy by interaction with LAMP2 (**Figure 1**), which modulates autophagosome-lysosome fusion (Costes et al., 2014; Hubert et al., 2016).

Uchl1 plays an important role in synaptic functions and memory as shown in mouse model of AD (Gong et al., 2006). This effect may be related to the Uchl1 ability to restore Bdnf signaling, which is disrupted by Aβ (Poon et al., 2013). BDNF is one of the most critical mediators of brain functions (Lu et al., 2014). Several publications have reported either effect or lack of effect of *UCHL1* mutations on AD (Xue and Jia, 2006; Shibata et al., 2012). Similarly, there is some discrepancy in the directionality of changes in expression of *UCHL1* gene between different studies performed on AD patients. In frontal cortices the UCHL1 protein was upregulated (Donovan et al., 2012). On the other hand, downregulation of UCHL1 was reported in hippocampi (Poon et al., 2013) and in unspecified brain area (Choi et al., 2004).

Co-immunoprecipitation assay showed that Uchl1 interacts with App (Zhang M. et al., 2014). The Uchl1 overexpression, induced by intracranial injection of *Uchl1*-expressing virus, decreases the Aβ production and protects AD model mice against memory impairment (Zhang M. et al., 2014). Decreased expression and activity of UCHL1 protein is associated with Aβ treatment *in vitro* (Guglielmotto et al., 2012). Similarly, decreased expression of UCHL1 protein is found in the cerebral cortex of AD patients (Guglielmotto et al., 2012). Additionally, the cortical UCHL1 protein levels seem to be inversely correlated to the number of NFT in AD patients (Chen et al., 2013). Moreover, UCHL1 is involved in lysosomal degradation of BACE1 (Guglielmotto et al., 2012).

UCHL1 protein co-localizes with NFTs in AD brains (Choi et al., 2004). The Uchl1 expression and activity negatively influence the levels of phosphorylated tau and aggregation of tau protein in mouse neuroblastoma cells (Xie et al., 2016). Tau induces mitochondrial degradation, synaptic deterioration, and cellular death by recruiting UCHL1 *in vitro* (Corsetti et al., 2015).

## Therapeutic Implications of the Interplay of Alzheimer’S Disease and Autophagy

The protein aggregates, e.g., Aβ and tau proteins, participating in the pathology of neurodegenerative disorders cause neuronal damage and synaptic dysfunction (Irvine et al., 2008; Bloom, 2014). Their removal or inhibition of their formation are proposed as potential therapeutic approaches for the treatment of neurodegenerative disorders (Nowacek et al., 2009). Autophagy is one of the main mechanisms by which the cell degrades abnormal proteins. Thus, elimination of such protein aggregates may be achieved utilizing mechanisms of autophagy (Metcalf et al., 2012). Several autophagy-stimulating drugs have already demonstrated considerable therapeutic potential for AD treatment in clinical trials. We shortly discuss some of them below.

### Carbamazepine (CBZ)

Carbamazepine was primarily developed as a drug used in the treatment of epilepsy (Okuma and Kishimoto, 1998). In the past, scientists studied therapeutic effect of CBZ on AD-related agitation (Xiao et al., 2010). Recently two publications have shown that carbamazepine-induced autophagy also protected against memory dysfunction and increase in Aβ content in brains of mouse model of AD (Li et al., 2013; Zhang et al., 2017).

### Latrepirdine

Latrepirdine stimulates mTOR- and Atg5- dependent autophagy and reduces intracellular content of App metabolites, including Aβ peptides, in the brain of mouse (Steele and Gandy, 2013). Recent meta-analysis has shown no adverse effects and small improvement in dementia-related behaviors by latrepirdine in AD patients (Chau et al., 2015). Nevertheless, as Chau et al. (2015) themselves admit, the analyzed literature was not comprehensive enough to allow for more confident conclusions.

### Lithium

Clinical trials have shown that lithium may ameliorate AD and this effect may be related to its mTOR-independent autophagy-inducing activity (Sarkar et al., 2005; Forlenza et al., 2012). In meta-analysis of clinical studies on AD, lithium significantly decreased cognitive decline compared to placebo, while showing no significant adverse effects (Matsunaga et al., 2015a).

### Memantine

The NMDA (*N*-methyl-D-aspartate) receptors antagonist memantine is widely used for treatment of moderate-to-severe AD. According to recent meta-analysis it shows good tolerance and some efficacy in AD treatment (Matsunaga et al., 2015b). This effect may be in some extent mediated by memantine ability to influence autophagy in either mTOR-dependent or mTOR-independent manner (Song G. et al., 2015).

### Nicotinamide

Liu et al. (2013) reported that long-term treatment with nicotinamide (Vitamin B3/PP) reduces Aβ and tau pathologies as well as cognitive decline in a mouse model of AD. The effect of nicotinamide is likely mediated by enhancement of the acidification of lysosome or autophagolysosome, leading to reduced autophagosome accretion (Liu et al., 2013). Gong et al. (2013) have shown that nicotinamide activity depends also on its ability to induce degradation of Bace1. Recently published clinical trials showed safety, but no effect of nicotinamide on cognitive function of AD patients (Phelan et al., 2017). Despite this, nicotinamide anti-AD activity is still studied and further trial is currently ongoing (Grill, 2017).

### Protein Phosphatase 2A Agonists

Clinical trials have suggested that protein phosphatase 2A agonists, such as metformin, can inhibit the hyperphosphorylation of tau (Kickstein et al., 2010). Similar results were obtained from a study on mice (Li et al., 2012). Hyperphosphorylation of tau is a key step in generation of NFTs in AD patients (Iqbal et al., 2010). On the other hand, metformin did not protect diabetic mice from AD-like memory dysfunction (Li et al., 2012).

### Rapamycin

Rapamycin, a selective inhibitor of target-of-rapamycin complex 1 (TORC1) and thus modulator of the mTOR pathway activity, improved learning and memory and reduced Aβ and tau pathology in the brains of AD mouse model (Caccamo et al., 2010; Spilman et al., 2010). Rapamycin also increased viability of cells treated with Aβ42 (Xue et al., 2013). Rapamycin prodrug, temsirolimus was shown to induce autophagy-dependent Aβ clearance and to improve memory in mouse model of AD (Jiang et al., 2014). Temsirolimus also lowered tau accumulation and rescued motor dysfunctions in tau mutant mice (Frederick et al., 2015). SMER28, a small molecule-based enhancer of rapamycin, increases autophagy via Atg5-dependent pathway while reducing the levels of Aβ peptide in a γ-secretase-independent manner (Tian et al., 2011). Recent rapamycin clinical trial showed non-significant decrease in expression of the cellular senescence marker beta galactosidase (Singh et al., 2016).

### Resveratrol

Resveratrol, a grape-derived polyphenol, and its derivatives decreased extracellular Aβ peptide accumulation by activating autophagy via AMPK signaling pathway (**Figure 1**) (Vingtdeux et al., 2010). Recently published clinical trials studying the efficacy of resveratrol for AD treatment showed that resveratrol is well-tolerated but, surprisingly, AD biomarkers, such as plasma Aβ40 level, were present in treated group at even higher levels than in a placebo group (Turner et al., 2015). On the other hand, long-term resveratrol treatment rescued memory loss and Aβ levels in the brain of AD mouse model (Porquet et al., 2014). Hence, viability of this compound as a medication for AD is unclear.

### Other Autophagy-Regulating Substances That Have Shown Relevant Results Only in Animal AD Models

#### Arctigenin

Arctigenin, a polyphenol extracted from *Arctium lappa*, was found to inhibit Aβ production and memory impairment in mouse model of AD (Zhu et al., 2013). The effect was mediated by mTOR- and AMPK-dependent autophagy (Zhu et al., 2013).

#### β-Asarone

β-asarone is an ether found, e.g., in *Acori graminei* (Liu et al., 2016). β-asarone treatment decreases Aβ42 levels in hippocampus and improves memory in a mouse model of AD, probably through mTOR-dependent autophagy (Deng et al., 2016).

#### GTM-1

It was shown that administration of GTM-1, a derivative of quinolone, rescues cognitive dysfunction and Aβ pathologies in mouse model of AD by activating mTOR-independent autophagy (Chu et al., 2013; Zhang et al., 2017).

#### Oleuropein Aglycone

Oleuropein aglycone is a polyphenol, which is present in plants of *Oleaceae* family and induces autophagy via mTOR pathway (Grossi et al., 2013; Luccarini et al., 2015). According to a recent review (Martorell et al., 2016), regulation of autophagy is one of the mechanisms via which oleuropein aglycone counteracts amyloid aggregation and toxicity.

#### Tetrahydrohyperforin

Tetrahydrohyperforin is a derivative of hyperforin, which is an active component of St. John’s Wort plant (*Hypericum perforatum*). In AD model mice tetrahydrohyperforin prevented memory impairment and physiological dysfunctions such as tau hyperphosphorylation or turnover of amyloid plaques (Cerpa et al., 2010; Inestrosa et al., 2011). At least one of its beneficial effects is mediated by its autophagy-related activity, that is clearance of APP via ATG5-dependent pathway (Cavieres et al., 2015).

#### Trehalose

The disaccharide trehalose, an inducer of mTOR-independent autophagy (Sarkar et al., 2007), inhibits the aggregation of both Aβ40 and tau, and reduces their cytotoxicity *in vitro* (Liu et al., 2005; Kruger et al., 2012). Similarly, in two separate studies utilizing mouse models of AD, trehalose protected against cognitive dysfunction (Du et al., 2013; Portbury et al., 2017). Interestingly, one of these studies also reported effect of trehalose on hippocampal Aβ levels (Du et al., 2013), while the other one reported a lack of this effect (Portbury et al., 2017).

Summarizing, scientific community puts a significant effort into developing autophagy-related therapeutics for AD. Several agents, such as rapamycin and latrepirdine, have already been tested on AD patients and show promising results. However, many more potential therapeutics showing efficacy for treatment of cognitive dysfunctions in animal models of AD await for more comprehensive studies and trials on humans.

## Conclusion

Despite much of the data presented in the review being acquired in studies performed on animal models, we propose that properly functioning autophagy is crucial for the normal aging of neurons. Malfunction in neuronal autophagy is one of the key factors influencing the development of neurodegenerative disorders, including AD. The autophagy plays a key role in the metabolism of Aβ and tau protein, the mTOR pathway, neuroinflammation, and in the endocannabinoid system, all of which may mediate its effect on AD. Accordingly, autophagy-targeted therapeutic approaches may lead to the development of novel therapeutic strategies for the management of AD.

## Author Contributions

This work was carried out in collaboration between all authors. MU, AMS, AS, and AM have written the first draft of the manuscript. NT, ST, AA, LB, and MA-D revised and improved the first draft. All authors have seen and agreed on the finally submitted version of the manuscript.

## Conflict of Interest Statement

The authors declare that the research was conducted in the absence of any commercial or financial relationships that could be construed as a potential conflict of interest.

## Abbreviations

Aβ: Amyloid β
AD: Alzheimer’s disease
CSF: cerebrospinal fluid
MAPT: microtubule-associated protein tau
NFTs: neurofibrillary tangles
